# Sliding Wear Behavior of Intermetallic Ti-45Al-2Nb-2Mn-(at%)-0.8vol%TiB_2_ Processed by Centrifugal Casting and Hot Isostatic Pressure: Influence of Microstructure

**DOI:** 10.3390/ma15228052

**Published:** 2022-11-15

**Authors:** Segundo Shagñay, Juan Cornide, Elisa María Ruiz-Navas

**Affiliations:** Material Science and Engineering Department-IAAB, University Carlos III of Madrid, Ave. Universidad 30, 28911 Leganés, Spain

**Keywords:** TiAl, reciprocating sliding, HIP, centrifugal casting

## Abstract

Intermetallic alloys such as titanium aluminides (TiAl) are potential materials for aerospace applications at elevated temperatures. TiAl intermetallics have low weight and improved efficiency under aggressive environments. However, there is limited information about wear behavior of these alloys and their microstructure. The present work aims to study the influence of the microstructure in the tribological behavior of TiAl intermetallic alloy (45Al-2Mn-2Nb(at%)-0.8 vol%TiB_2_). Wear tests were performed on samples manufactured by centrifugal casting (CC) and hot isostatic pressure (HIP). Reciprocating sliding wear test was carried out for TiAl, it was combined with different loads and frequencies. Wear tracks were analyzed through opto-digital microscopy and electron microscopy (SEM). The results obtained reveal that CC intermetallics present the lowest volume wear lost, approximately 20% less than HIP intermetallics. This good behavior could be related to the high hardness material, associated with the main microstructure where CC intermetallic has nearly lamellar microstructure and HIP intermetallics present duplex microstructure.

## 1. Introduction

The TiAl intermetallic alloys are attractive and lightweight with good physical and mechanical properties at high temperatures. Moreover, the low density [1] and good corrosion resistance [2] make them ideal for structural applications in many areas such as aerospace, military, sport equipment, chemical engineering, and automotive industry [3,4,5]. In addition, TiAl intermetallics represent one third of the weight of modern aircraft engines and TiAl are the second most used materials following Ni-based superalloys in airspace industry. The first commercial use of TiAl intermetallic alloys was in high performance turbochargers for Formula 1 and sports cars [6].

CC and HIP are the two processes by which this type of intermetallic alloy can be manufactured. CC uses using a ceramic mold coated with Y_2_O_3_ under the following conditions: mold rotation velocity of 250 rpm, mold preheating temperature of 1200 °C. After casting, the alloy was processed by HIP at 1260 °C and 140 MPa for 4 h in order to remove any remnant porosity [7]. On the other hand, the powder metallurgy specimens are manufactured by HIP of pre-alloyed powder. The powders were obtained by electrode induction gas atomization (EIGA) and processed by HIP at 1200 °C and 200 MPa for 4 h under Argon atmosphere [8].

Currently, CC and HIP processes are used to manufacture the TiAl intermetallic alloys due to CC and HIP being routes at high temperature, through which it allows decreased costs by avoiding material losses and final machining that is generated with traditional techniques [9]. The main advantage of the CC process is the variation of key parameters such as rotation or pour speed preventing the formation of pores inside the material [10]. Through this technique, the structures obtained are lamellar and duplex and some pieces produced by this technique are turbine vanes and valves for the automotive industry [7,11]. On the other hand, the HIP technique is a well stabilized and consolidated powder metallurgy technique; through this technique, the microstructure of TiAl is typically duplex with gamma grains and laminar grains and this kind of intermetallic is used to manufactured important pieces of the Tren 1000 engine [4].

However, some authors [1,12,13] have reported that the mechanical properties of the TiAl intermetallic depend mainly on their microstructure. Then, fully lamellar microstructure has high strength and creep resistance, while duplex microstructure is softer but possesses higher room temperature elongation to failure. Finally, there are other factors to be considered; for the mechanical properties regarding microstructure, it should be: interface type distribution, lamellae orientation, and grain size. 

From the point of view of the mechanical properties, wear and/or abrasion resistances of TIAl intermetallic materials are of great interest [14,15]. The wear behavior is important for understanding problems such as the wear of surface (turbine vanes) or surface problems due to scratching in pieces of the Tren 1000 engine [4]. There are different techniques to perform analyses of wear of different types of surfaces such as dry sliding wear and/or pin on slab as described Fernandez et al. [16] and Saldívar et al. [17], respectively. 

Rastkar et al. [18] studied the wear behavior of a TiAl intermetallic alloy using the pin on disk technique; they demonstrated that abrasive wear causes a slight deformation of lamellar and slip through the interlamellar. Moreover, the high hardness and the reduction of the grain size are important factors in determining the wear resistance of the TiAl [19,20,21,22].

Okonkwo et al. [23] determined that the effect of temperature of the surface is an important key to determine the wear behavior due to the temperature generated and it could cause or increase the severity of wear mechanism on the sliding surfaces. In addition, temperature in the wear test due to friction could exceed 130 °C [24]; it directly affects the microstructure behavior of the intermetallic.

Therefore, the aim of this work is to study the wear behavior of the Ti-45Al-2Mn-2Nb(at%)-0.8vol%TiB_2_ (Ti4522XD) by reciprocating sliding wear test to evaluate their wear behavior. The samples under study were processed by CC and HIP techniques and these were tested under different load and frequency conditions. Finally, the differences of the worn surfaces, the coefficient of friction, and mass loss were determined.

## 2. Materials and Methods

TiAl intermetallic alloy with a nominal composition of Ti-45Al-2Nb-2Mn(at%)-0.8vol%TiB_2_ (Ti4522XD) [1] obtained by CC and HIP processes were used to perform the wear test. The specimens of Ti4522XD alloy were obtained measuring 1 × 2 × 0.5 cm suitable for the reciprocating wear equipment [7]. First of all, in order to keep the same surface conditions, all the samples were ground and polished up to 1 µm with diamond paste according to standards ASTM E3. Kroll (4 vol% HF + 10 vol% HNO_3_ + 86 vol%H_2_O) was used as an etching solution to reveal the microstructure [25]. 

The microstructural characterization and chemical composition were performed by a scanning electron microscope (SEM) with an Energy Dispersive Spectroscopy (EDS) detector Octane Plus (TENEO FEI, Eindhoven, The Netherlands). In addition, Vickers micro-hardness tests were also performed to determine hardness difference between samples with CC and HIP processes it was processed by means of micro-hardness instrument (Zwick Roell, Ulm, Germany) using 500 gf load and dwell time of 10 s for each point, and the data of the eight points per sample were analyzed by the hardness testing software ZHµHD. Measurements were carried out on the surface to be exposed to wear test. Finally, the roughness was measured by means of a linear contact roughness meter (Höhenstander HS-305, Villingen-Schwenningen, Germany) with a software that allows to determine roughness by difference of depth where difference of depth value refers to the roughness of the surface [26].

Reciprocating sliding wear test was carried out with an UMT-TriboLab CETR-UMT and CETR-APEX TriboLab equipment (Mannheim, Germany). The test was developed under dry conditions and room temperature. The counterbody was an Al_2_O_3_ ceramic ball with a diameter of 5 mm (with ± 2.5 μm tolerance). All the wear tests were developed for 30 min with amplitude of the wear track of 5 mm. However, different loads and frequencies were selected as can be seen in Table 1. The tests with each one of the combinations were carried out at least three times.

**Figure 1:**
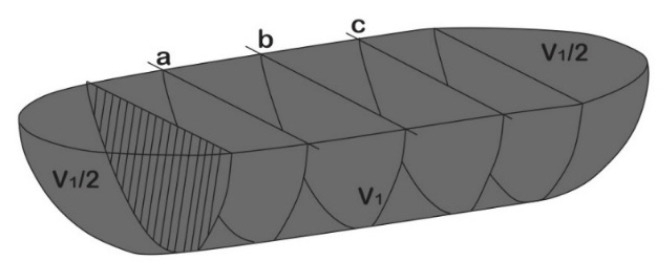
Model for the calculation of the lost volume after the wear test [27].

The coefficient of friction curves versus sliding time were automatically recorded by the UMT testing software Viewer. In addition, the morphology and volume of the wear tracks was characterized by an opto-digital Olympus (DSX500, microscope, Tokio, Japan). The volume of the material lost was estimated according to Figure 1 [27], using the Archard equation shown in Equation (1) for the calculation of the worn volume.

(1) $$\Delta V=\left[1/3*\pi *\bar{D^{2}}\left(3R-\bar{D}\right)\right]+\bar{A_{w}}*l$$

where Δ*V* is the total volume loss in mm^3^ for each wear track, $\bar{D}$ is the average depth in mm, *R* is the radius of the counterbody, in this case is the alumina ball with 5 mm diameter, $A_{w}$ is the average wear loss area of three 2D profile in mm^2^ for each wear track, and *l* is the total stroke length which was constant for all tests (5 mm) [27].

Moreover, after wear tests the wear tracks were characterized using a SEM Philips XL-3 microscope, which incorporates an EDAX DX-4 detector. Each wear track was analyzed trying to understand the influence of testing parameters as well as microstructural features on wear behavior of the material. 

## 3. Results and Discussion

### 3.1. Microstructural Characterization

**Figure 2:**
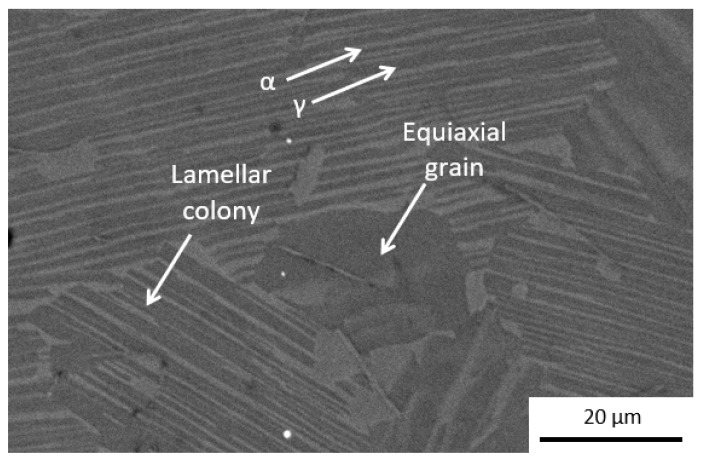
Representative microstructure of Ti4522XD-CC, showing the different areas analyzed.

The microstructure of the samples Ti4522XD obtained by CC is observed in Figure 2. Figure 2 clearly consists of a nearly lamellar microstructure, which is formed by a minor fraction of equiaxial grains embedded in a matrix of lamellar colonies; moreover, these lamellar colonies are formed by alternate sheets of α (Ti_3_Al) and ɣ (TiAl) phases colonies. This type of microstructure is typically observed for this Ti4522XD intermetallic with the same composition and similar heat-treatments [3,8,28]. Furthermore, EDX analyses performed in each region (lamellar colony and equiaxial grain) from Figure 2 are summarized in Table 2. The results show that the region of equiaxial grains encompasses an area rich in Al and Ti, while regions of lamellar colonies present higher Ti content.

**Figure 3:**
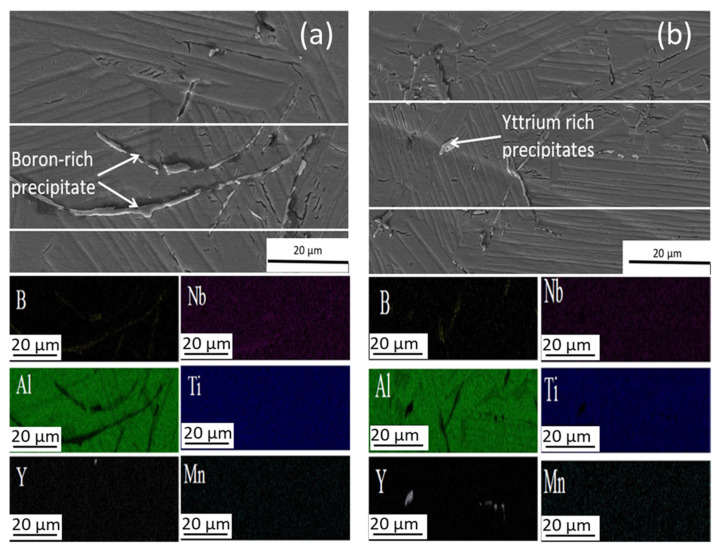
Details of microstructures of Ti4522XD-CC (**a**) boron-rich precipitates, and (**b**) yttrium-rich precipitates and EDAX mapping analysis of precipitates.

Figure 3a,b shows the representative area of the CC samples with the respective EDAX mapping analysis. Precipitates with different shapes can be seen, acicular or needle-like (Figure 3a) and polygonal-like (Figure 3b). According to the EDX analysis, the acicular precipitates are boron-rich (Figure 3a) [3,8] and polygonal-like precipitates are rich in yttrium content (Figure 3b). The presence of yttrium can only be explained by the contamination from the mold walls where centrifugal casting was done [8] and as these yttrium-rich precipitates are similar to the Y_2_O_3_ observed by Moreno et al. [1]. Regarding the main components, Ti, Al, and Nb are homogeneously distributed in the matrix according to the element distribution map (Figure 3).

**Figure 4:**
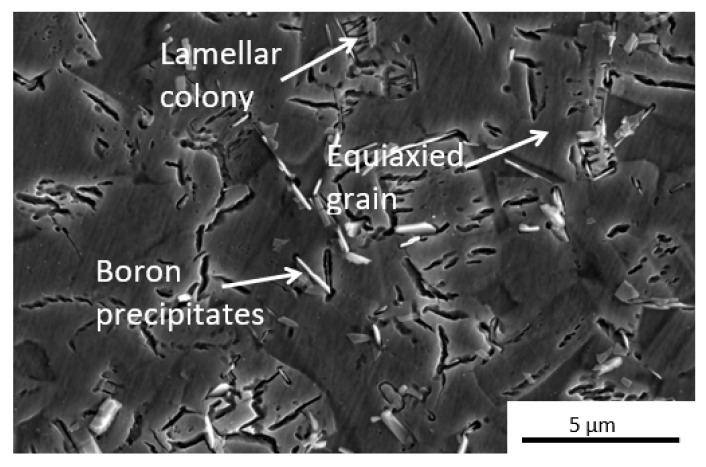
Representative microstructure Ti4522XD-HIP obtained from SEM.

The microstructural features observed in Figure 4 belong to Ti4522XD-HIP samples. As explained above, the HIP process allows to obtain duplex microstructure [1,29]. This type of microstructure is formed by small ɣ equiaxed grains and lamellar colonies. The equiaxial grains present a size approximately of 5 µm similar to lamellar colonies [1,8]. In addition, small banded-shape precipitates are homogeneously distributed in the matrix with not preferential orientation.

**Figure 5:**
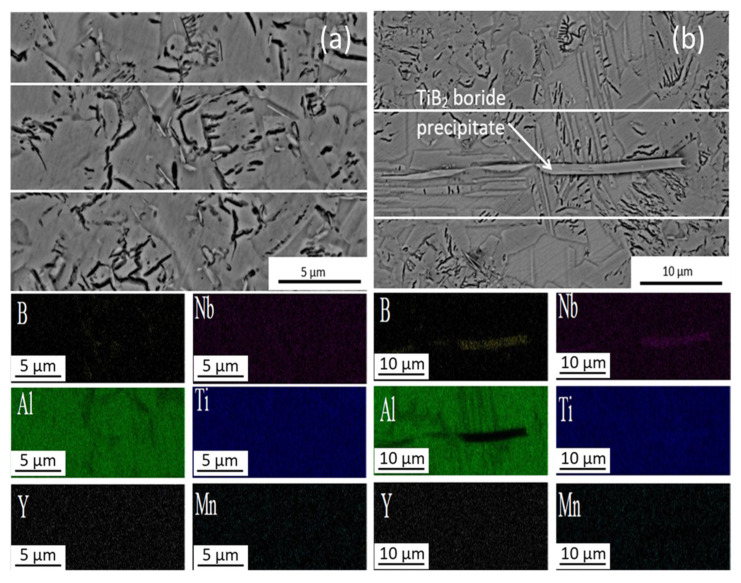
Details of microstructures of Ti4522XD-HIP (**a**) boron-rich precipitates, and (**b**) TiB2 precipitates and EDAX mapping analysis.

Figure 5a,b show a representative area of Ti4522XD-HIP samples and the respective EDAX mapping analysis. It can clearly be seen that Al, Ti, Mn, and Nb are homogeneously distributed in the matrix. In addition, Figure 5b shows trans granular band-shaped precipitates; these precipitates cross the ɣ equiaxed grains and lamellar colonies, which fits the composition of TiB_2_ borides and these borides are similar to those observed in Ti4522XD-CC samples whose size is around 25 µm. Previous studies [3,28] revelated that band-shaped borides are formed during slow cooling with a minimum of 0.5 at.% of B-content. Furthermore, these borides lead to premature failure when the material is exposed to high mechanical stresses [28].

### 3.2. Hardness Characterization

Table 3 details the Vickers hardness of both materials before the dry sliding wear test. From Table 3 results, Ti4522XD-HIP samples (duplex microstructure) present lower hardness than Ti4522XD-CC samples (nearly lamellar microstructure). Cheng et al. [30] demonstrated that nearly lamellar microstructure has better resistance to being penetrated than Ti4522XD-HIP materials. Moreover, the high hardness of the nearly lamellar samples is related to those boron-rich and yttrium-rich precipitates.

### 3.3. Roughness

**Figure 6:**
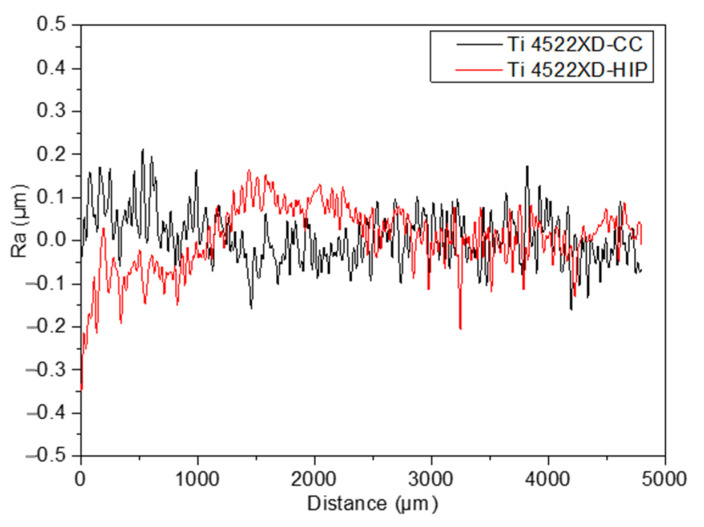
Example of roughness profile for Ti4522XD-CC and Ti4522XD-HIP samples.

According to ISO 14577-1 standard, the surface of the specimens has a significant influence on the wear behavior. Thus, Figure 6 shows the roughness of two types of samples under study. In this case, the arithmetic mean roughness (Ra) was measured, values being the average of 30 measurements. As can be seen in Figure 6, the roughness profile for TiAl alloys processed by CC and HIP despite the small differences in the initial steps between the two samples, according to the difference of the roughness along the whole 500 μm, can be considered that both have the same roughness behavior. In accordance with this assertion, Table 4 shows the measured Ra average.

### 3.4. Reciprocating Sliding Wear Test

#### 3.4.1. Coefficient of Friction

**Figure 7:**
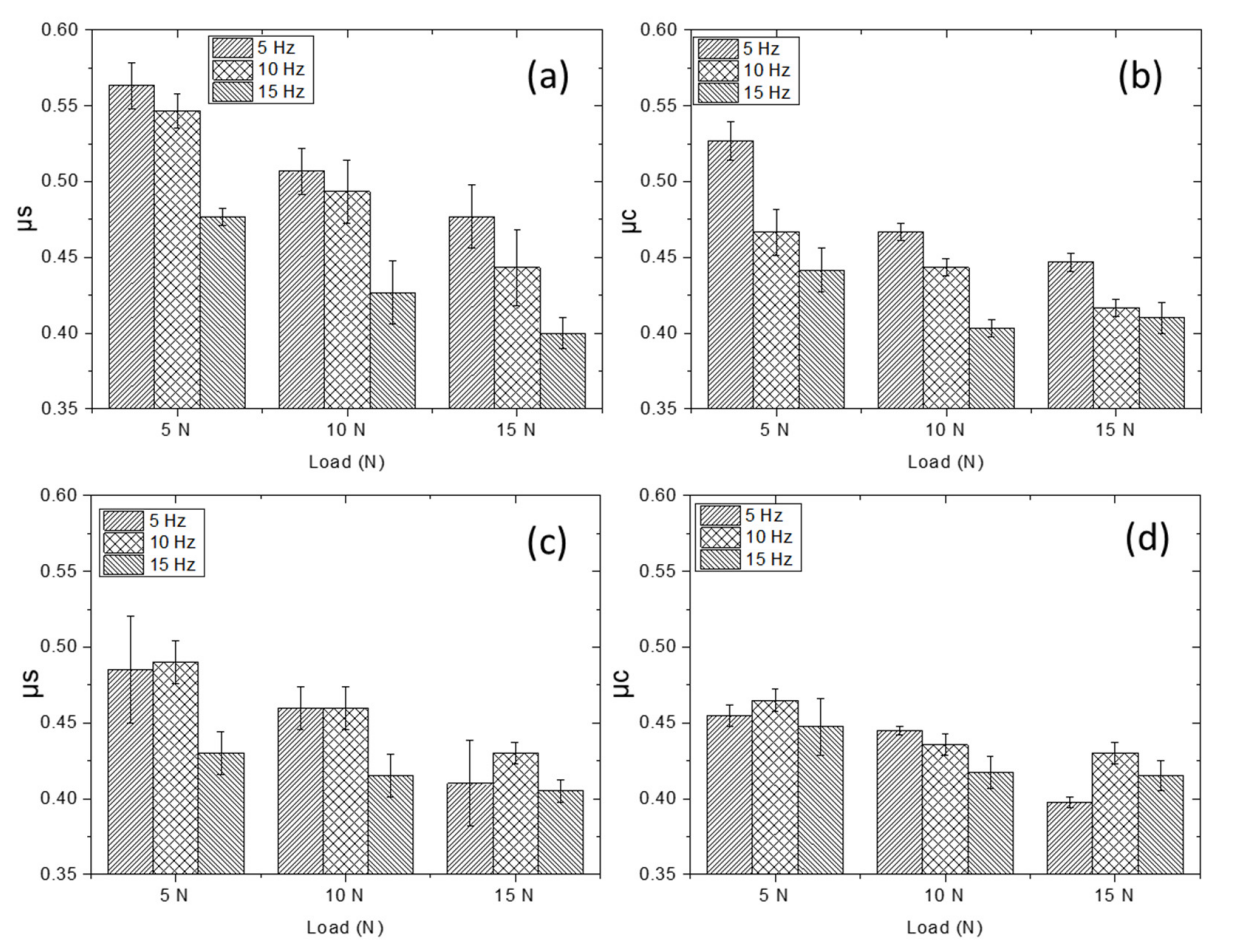
(**a**) µs, (**b**) µc of Ti4522XD-CC, and (**c**) µs (**d**) µc Ti4522XD-HIP.

Figure 7 shows the results of the static (µs) and dynamic (µc) coefficient of friction (COF) obtained from wear test. The average of µs and µc was determined from the maximum point of COF curve where it is stabilized [31]. As can be seen, the values of µs and µc for both materials decrease regarding to the load and frequency.

There is a small difference between the µs and µc of Ti4522XD-CC and Ti4522XD-HIP samples. As expected, values for µs are usually higher than those obtained for the µc [31]. Furthermore, it can be observed that the µs and µc of Ti4522XD-CC samples are higher than those of Ti4522XD-HIP samples for the three different conditions. This difference is due to the higher hardness of the alloys when those are processed by Ti4522XD-CC compared to the alloy processed by HIP [3,8].

Liu et al. [32] studied the relationship between the hardness and wear and they determined that COF decreases regarding the hardness. However, it can be observed that the results are opposite to expected as those samples with a lower hardness show higher μs values. This phenomenon can only be explained by the role that microstructure featuring plays in final material properties and the presence of precipitates [18,21]. As can be seen in Figure 5, the materials of Ti4522XD-HIP have larger precipitates than Ti4522XD-HIP materials.

#### 3.4.2. Wear Track Analysis and Volume Lost

**Figure 8:**
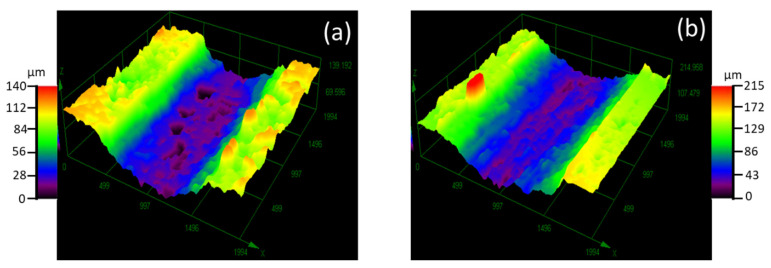
3D opto-digital images of representative wear tracks (**a**) Ti4522XD-CC and (**b**) Ti 4522XD-HIP.

**Figure 9:**
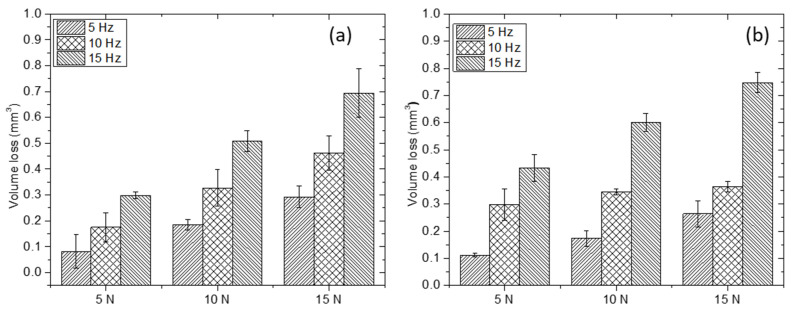
Volume loss (**a**) Ti4522XD-CC, and (**b**) Ti4522XD-HIP material.

Figure 8 shows examples of 3D images of representative wear tracks, after the reciprocating wear test for two type of samples. Differences in the depth and width of the wear tracks due to the composition of the Ti4522XD intermetallic can be seen in Figure 8. The change in these dimensions is used for calculating the volume losses plotted in Figure 9.

It can be appreciated that the Ti4522XD-CC present the smaller and least deep wear track for all test condition. In addition, when the frequency increased to 15 Hz under a load of 15 N, the depth and the width of wear tracks exhibited a higher difference between the Ti4522XD-CC and Ti4522XD-HIP materials. This is due to the high hardness of Ti4522XD-CC versus to low hardness of Ti4522XD-HIP.

On the other hand, there are some holes inside of the wear track (dark areas in Figure 8a,b) in the same direction of the counterbody sliding. Shaik et al. [33] and Yi, et al. [34] determined that those holes are due to abrasion of harder particles such as boron particles on the surface promoting abrasive wear. Therefore, as can be seen in Figure 8 there are bigger holes in Ti 4522XD-CC despite this material having a small wear track. These observations lead to the results in Figure 9; as expected, the volume loss in both materials Ti4522XD-CC and Ti4522XD-HIP increased in terms of the load and frequency.

It must be pointed out that the volume loss in Ti4522XD-CC material is lower than that of the Ti4522XD-HIP material. It is directly explained by the main wear mechanism, the different microstructural features (as mentioned above), and the hardness difference (seen Table 4). Consequently, Ti4522XD-HIP material showed severe damage of the surface and higher material loss. On the other hand, the Ti4522XD-CC material showed lower damage in wear surface, and it corresponds to a lower material loss after wear test.

#### 3.4.3. Wear Track Analyses by SEM

**Figure 10:**
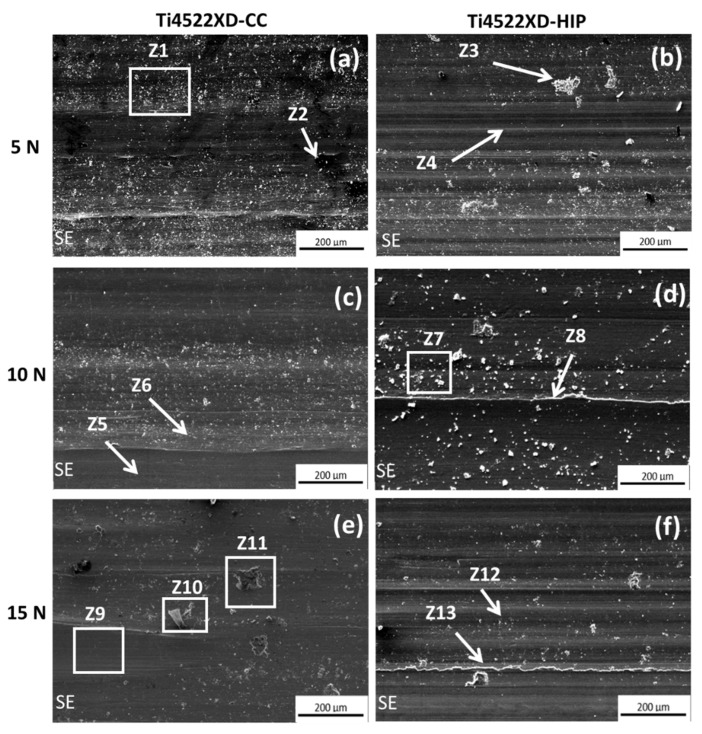
Representative areas of the wear tracks with different loads for 15 Hz of frequency. (**a**,**c**,**e**) Ti4522XD-CC and (**b**,**d**,**f**) Ti4522XD-HIP.

Figure 10 shows representative SEM images of the microstructures of worn surfaces of the Ti4522XD-CC and Ti4522XD-HIP samples. From this image, the main effect on wear tracks surface of both materials was the accumulation of debris its accumulation of debris increases regarding to load and frequency. From the wear tracks, 13 representative areas (Z1 to Z13) were selected in order to study different microstructure features. Then, Z1, Z3, Z7, Z10, and Z11 areas showed clearly a high debris accumulation. Yi et al. [34] determined that debris accumulation typically is due to abrasion wear on material surfaces. In addition, Table 5 shows the chemical composition of some interesting areas and debris on the surface after wear test. From the results of Table 5, it can be observed that there is high content of oxygen, mainly due to surface oxidation [30,35]. Moreover, the debris are composed of a high content of Al and Ti, which is associated with the composition of the raw material. In addition, the high percentage of Al may be associated to raw material; moreover, it can be derived from the counterbody that it is composed by Al_2_O_3_.

On the other hand, wear track surfaces present delamination, as can be observed in Z2 zone (Figure 10a). Prabhu et al. [35] and Manohara et al. [36] mentioned that delamination is generated by effect of adhesive wear either by harder particles that accumulate on the surface or by the counterbody. Moreover, the wear tracks show grooves accumulation in regions as Z4 and Z12 (Figure 10b,f respectively); these grooves are a consequence of abrasion wear according to Shaik et al. [33]. Finally, microcutting can also be generated by action of the counterbody on the material surface. Yi et al. [34] mentioned that the formation of lines in the same direction of counterbody reveal the existence of the microcutting in the microstructure.

Nevertheless, Figure 10c shows two areas with different wear behavior (Z5 and Z6), Z5 shows a clean area without cracks or groves; it can be observed that adhesion wear is occurring. Meanwhile, zone Z6 shows the presence of debris on all surface, which is generated by abrasive wear. Zones Z8 and Z13 of the Figure 10d,f, respectively, show grooves in the same sliding direction with accumulation of debris on edges. Yang et al. [37] mentioned that those grooves are due to plastic deformation on surface.

Figure 10e, zone Z9, exhibits an area with macro grooves in the same sliding direction of the counterbody. As mentioned above, these macro grooves are mainly due to abrasive wear and it leads to a delamination of the surface material.

To summarize, Ti4522XD-HIP material shows more damage to the surface relative to Ti4522XD-CC material. As mentioned previously, Ti4522XD-HIP materials have a larger wear track than Ti4522XD-CC (Figure 8). In addition, Ti4522XD-HIP materials have a duplex microstructure and they exhibit higher plastic deformation when subjected to wear test and suffer higher loss of material by effect of wear. Nevertheless, both Ti4522XD-CC and Ti4522XD-HIP materials present abrasion and adhesion wear, the abrasion wear being the predominant wear mechanism for both materials.

## 4. Conclusions

In this work, TiAl intermetallic alloys manufactured by centrifugal casting and hot isostatic pressure techniques were used to study wear behavior. After carrying out this study, the following main conclusions can be drawn:

Ti4522XD-CC and Ti4522XD-HIP materials show nearly lamellar and duplex microstructure, respectively. The microstructure plays a main role in the properties such as hardness where nearly lamellar shows higher hardness than duplex microstructure.

Ti4522XD-CC material was the most effective against wear. This good performance is related to nearly lamellar microstructure. Moreover, this helps us to understand the good performance of nearly lamellar microstructure, which is able to withstand wear stresses. On the other hand, the Ti4522XD-HIP material was least effective against wear behavior.

Abrasive and adhesive wear were found in both materials. SEM images detail the effect of wear on the surface of the material where debris, grooves, and delamination zones were observed. Finally, abrasive wear is predominant in both types of materials.

## Figures and Tables

**Table 1:** Loads and frequencies used for wear test.

Load (N)	Frequency (Hz)
5	5
	10
	15
10	5
	10
	15
15	5
	10
	15

**Table 2:** Chemical composition of CC samples of different zones from Figure 2.

Material	Area	Al (wt%)	Nb (wt%)	Ti (wt%)	Mn (wt%)
Ti4522XD-CC	Equiaxial grain	32.9	4.9	58.6	3.3
	Lamellar colony	26.5	4.2	65.8	3.4

**Table 3:** Vickers hardness for Ti4522XD obtained by CC and HIP.

Sample	Hardness (HV 0.5)
Ti 4522XD-CC	400 ± 20
Ti 4522XD-HIP	357 ± 18

**Table 4:** Roughness average (Ra) of Ti4522XD-CC and Ti4522XD-HIP samples.

Sample	Roughness Ra (μm)
Ti 4522XD-CC	0.06 ± 0.03
Ti 4522XD-HIP	0.06 ± 0.02

**Table 5:** Chemical composition of different areas of wear tracks from Figure 9.

Sample	Area	Al (wt%)	Nb (wt%)	Ti (wt%)	Mn (wt%)	O (wt%)
Ti4522XD wear track	Z1	30.1	4.6	57.3	3.5	4.6
	Z3	28.6	3.8	55.0	3.0	9.7
	Z7	31.2	4.7	58.3	2.9	2.8
	Z10	32.7	5.4	52.5	2.8	6.5
	Z11	33.5	3.9	54.9	3.0	4.7

## Data Availability

Not applicable.
